# An Overview for Clinicians on Intraductal Papillary Mucinous Neoplasms (IPMNs) of the Pancreas

**DOI:** 10.3390/cancers16223825

**Published:** 2024-11-14

**Authors:** Dimitrios Moris, Ioannis Liapis, Piyush Gupta, Ioannis A. Ziogas, Georgia-Sofia Karachaliou, Nikolaos Dimitrokallis, Brian Nguyen, Pejman Radkani

**Affiliations:** 1 MedStar Georgetown Transplant Institute, Washington, DC 20007, USA; piyush.gupta@medstar.net (P.G.); brian.m.nguyen@gunet.georgetown.edu (B.N.); pejman.radkani@gunet.georgetown.edu (P.R.); 2 Department of Surgery, University of Alabama at Birmingham, Birmingham, AL 35294, USA; iliapis@hsph.harvard.edu; 3 Department of Surgery, University of Colorado School of Medicine, Aurora, CO 80045, USA; iaziogas.md@gmail.com; 4 Division of Gastroenterology, Department of Medicine, Duke University Medical Center, Durham, NC 27710, USA; y.karachaliou@hotmail.com; 5 1st Department of Surgery & Organ Transplant Unit, Evangelismos General Hospital, 10676 Athens, Greece; nikolaosdimitrokallis@gmail.com

**Keywords:** intraductal papillary mucinous neoplasms, pancreatic cancers, biomarkers

## Abstract

Currently, there is no reliable method of discerning between low-risk and high-risk intraductal papillary mucinous neoplasms (IPMNs). Operative resection is utilized in an effort to resect those lesions with high-grade dysplasia (HGD) prior to the development of invasive disease. The current guidelines recommend resection for IPMN that involve the main pancreatic duct. Resecting lesions with HGD before their progression to invasive disease and the avoidance of resection in those patients with low-grade dysplasia is the optimal clinical scenario. Therefore, the importance of developing preoperative models able to discern HGD in IPMN patients cannot be overstated. Low-risk patients should be managed with nonsurgical treatment options (typically MRI surveillance), while high-risk patients would undergo resection, hopefully prior to the formation of invasive disease. Current research is evolving in multiple directions. First, there is an ongoing effort to identify reliable markers for predicting malignant transformation of IPMN, mainly focusing on genomic and transcriptomic data from blood, tissue, and cystic fluid. Also, multimodal models of combining biomarkers with clinical and radiographic data seem promising for providing robust and accurate answers of risk levels for IPMN patients.

## Simple Summary

This review specifically examines the advancing comprehension and management of intraductal papillary mucinous neoplasms (IPMNs) by integrating various diagnostic approaches, encompassing genomic and molecular biomarkers, alongside clinical and radiographic information. This review highlights the difficulty in differentiating low-risk from high-risk IPMNs and underscores the significance of biomarkers such as KRAS and GNAS mutations, which are directly linked to IPMN progression. Furthermore, it examines the efficacy of advanced imaging modalities, including MRI with MRCP and contrast-enhanced endoscopic ultrasound (EUS), for enhanced malignancy detection. This review also emphasizes novel models that integrate cystic fluid markers, clinical nomograms, and radiomics to enhance risk stratification. This review is unique in that it provides a thorough analysis of new molecular insights and emphasizes customized patient care while providing useful suggestions based on current international guidelines. This review helps clinicians improve IPMN treatment strategies and outcomes.

## 1. Introduction

Owing to the availability of high-quality imaging in our era, the frequency of pancreatic cystic neoplasm diagnosis has increased, with incidental cyst prevalence as high as 13.5% on magnetic resonance (MR) imaging [1]. Overall, more recent evidence places the prevalence of IPMN at around 10%, using CT scans as reference given its popular use in the United States [2]. Intraductal papillary mucinous neoplasms (IPMNs) represent the majority of neoplastic cystic lesions and are of particular importance to surgeons as these lesions are considered to be precancerous. They are cystic neoplasms originating from the epithelial cells of the pancreatic ducts. Their main characteristic is cell proliferation in the form of dysplastic papillary projections and increased mucin secretion that leads to cystic dilatation [3]. IPMN is a relatively new disease, since it was first described in 1982, even though there are data supporting its existence before the 1980s [4,5,6,7]. The World Health Organization (WHO) introduced IPMNs as a separate entity from mucinous cystic neoplasms in 1996 [7]. Initially, IPMN was considered a rare disease since a small number of cases were published up until 2000 [8]. However, this number increased over the following years, most likely due to an increasing number of imaging examinations performed [9], an increasing elderly population, and an increasing number of pancreatic cyst resections [10].

Despite therapeutic advancements, the prognosis for pancreatic cancer remains poor [11]. Theoretically, the resection of pre-malignant pancreatic cystic lesions is the best strategy for preventing malignant progression to pancreatic adenocarcinoma (PDAC) and thus improving patient survival. The latter demonstrates a pathway of progression from low-grade to high-grade dysplasia and eventually invasive adenocarcinoma. Currently, the clinical management of IPMN relies on several guidelines based on laboratory, endoscopic, cytologic, and imaging findings to properly select patients for surgery [12,13]. Accurate diagnosis of malignant precursors is needed to prevent patients with IPMN from being exposed to unnecessary surgery and its related morbidity and mortality. Advances in current diagnostic modalities and ongoing research on identifying biomarkers will facilitate the reliable discrimination of malignant from benign pancreatic cystic lesions. Among the most prevalent pancreatic cystic lesions, IPMNs have the greatest risk for malignant transformation, and this is why they should be investigated more in order to mitigate the grievous outcomes of PDAC [14]. However, the wide disease spectrum as well as the heterogeneity of the risk for malignancy pose a great challenge in the management of IPMN, which has prompted extensive research on biomarkers to better predict its pathologic behavior.

In this review, we summarize current understandings of the diagnostic strategies and highlight the most recent advancements in the clinical management of IPMNs.

## 2. IPMN Classification and Characteristics

### 2.1. Anatomic and Histologic Classification

**Figure 1:**
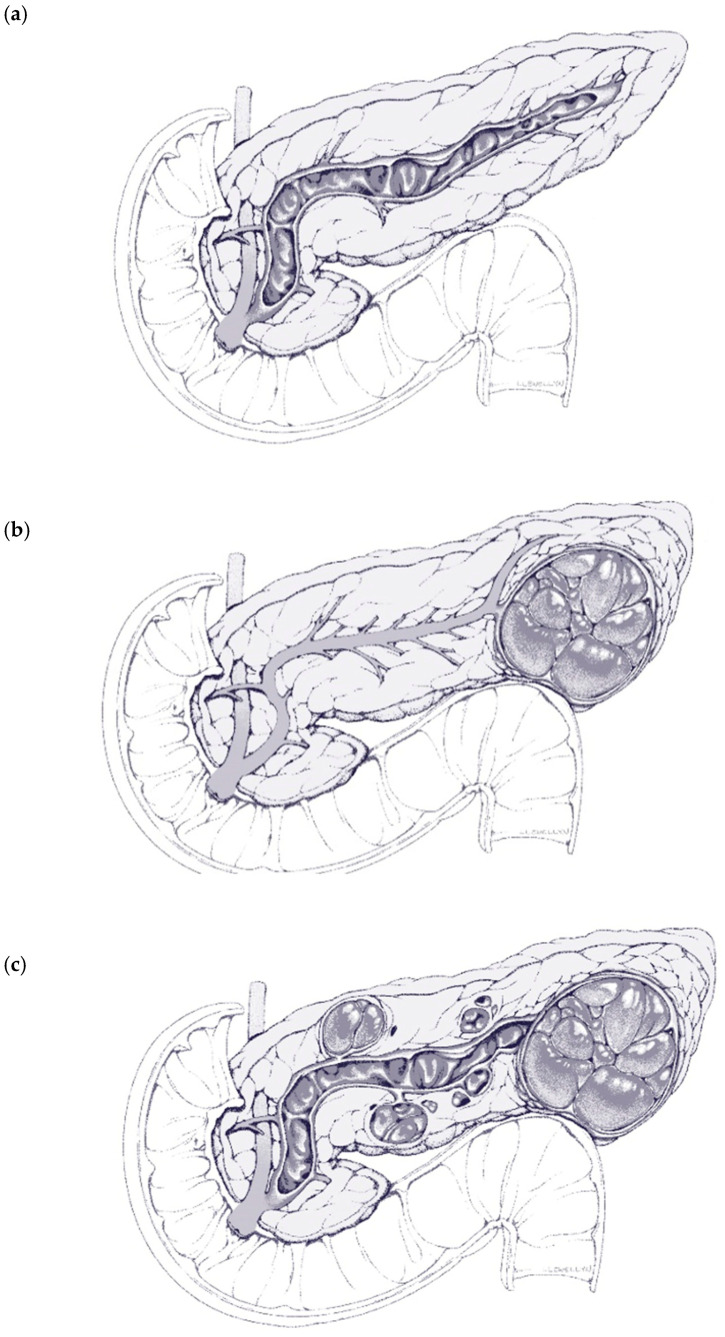
Anatomic subtypes of IPMN: (**a**) main duct dilatation, (**b**) side branch duct dilatation with mass, (**c**) mixed type—main duct dilatation with masses arising from side branches.

The anatomical categorization of IPMNs is threefold and based primarily on their location relative to the pancreatic ducts. Namely, the main three categories are main duct (MD) IPMNs, branch duct IPMNs, and mixed (MT) IPMNs (Figure 1a–c). Regarding their histological features, they are subdivided into four categories with varying dysplasia: gastric, intestinal, pancreatobiliary, and oncocytic [15,16]. The main characteristic of MD-IPMN is the prominent dilation of the main pancreatic duct; many cases present a bulging ampulla-secreting thick mucin (‘fish-eye’), a pathognomonic feature for this type. On the other hand, BD-IPMN presents as a dilation of the branches of the main pancreatic duct, usually with a ‘grape-like’ appearance. Lastly, the mixed type satisfies the criteria of the two aforementioned types.

In general, IPMNs can occur in any part of the pancreas, with the head being the most common location (70%), followed by the body/tail and multifocal occurrences, with 20% and 5–10% frequencies, respectively [16,17,18]. Since the management of IPMN depends and varies based on its type, the distinction between the different subtypes is of paramount clinical importance. The current work-up of a newly diagnosed IPMN includes CT, enhanced MRI with MRCP [19], and, if indicated, endoscopic ultrasonography (EUS) [20]. EUS may be used as an adjunct to other imaging modalities in cases where pancreatic cystic neoplasms show worrisome features or high-risk stigmata (mural nodules, dilatation of the pancreatic duct, or a thickened enhancing wall). Also, cyst fluid analysis for cytology and biochemical analysis can facilitate the differential diagnosis and elucidate the nature of the lesion, thus affecting patient management [20].

### 2.2. Radiographic Classification

Radiographically, worrisome (≤5 mm) and high-risk (>5 mm) enhancing mural nodules (unadjusted OR of 20 and 25, respectively, *p* < 0.001), abrupt main pancreatic duct size changes (unadjusted OR = 18, *p* < 0.001), lymphadenopathy (unadjusted OR = 7, *p* = 0.006), increasing main pancreatic duct size in mm (unadjusted OR = 1.1, *p* = 0.003), and cyst growth rate of the lesion in mm per year (unadjusted OR = 1.3, *p* = 0.04) are indicative of malignant nature in IPMNs [21]. Ohno et al. also supported the fact that, apart from the nodule size, the presence of enhancing solid components are highly associated with potential malignant transformation. Therefore, contrast-using radiographic tools seem to have better accuracy in their predictions [22]. Other predictors for malignancy in the BD-IPMN are jaundice, pancreatic head tumors, main pancreatic duct size > 5 mm, mural nodule size > 5 mm, CA19-9 level, positive cytology, and a CEA level > 30 ng/mL. In multivariate analysis, a mural nodule size > 5 mm and a CEA level > 30 ng/mL proved to be independent factors associated with the development of malignancy. The positive predictive value (PPV) of a mural nodule with a size > 5 mm and a CEA level > 30 ng/mL was 100 [23]. MRI with MRCP is considered the preferred follow-up method in cases for IPMNs, since it shows greater sensitivity compared to CT in identifying communication with the pancreatic ducts and the presence of high-risk stigmata and worrisome features [24]. There are several studies published in the medical literature that have tried to identify negative prognostic factors for the malignant transformation of IPMNs. In a large study of 1036 cases of BD-IPMNs, only 4.2% of the patients developed worrisome features/high-risk stigmata during a median follow-up of 62 months, whereas only 1.1% of them developed PDAC after a median time interval of 62 months. A growth rate ≥ 2.5 mm/year and the development of worrisome features were found as independent negative factors associated with the transformation to PDAC [25]. In the same vein, the standardized incidence ratio of PDAC for BD-IPMN was 22.45, which decreased to 3.84 when considering only patients older than 65 years. The authors concluded that surveillance of the vast majority of presumed BD-IPMNs is considered a safe practice, as the risk for malignant transformation is comparable to postoperative mortality rates after pancreatic surgery [25]. Similarly, Oyama et al. followed BD-IPMN patients for 15 years, finding a PDAC incidence of 3.3%, 6.6%, and 15.0% at 5, 10, and 15 years. In patients under surveillance for over 5 years, the total rates of PDAC were 3.5% and 12% after 10 and 15 years, respectively, from the first diagnosis. The IPMN size and main pancreatic duct diameter were linked to the development of IPMN-derived carcinoma (SHR 1.85; 95% confidence interval 1.38–2.48 for a 10 mm increase in the IPMN size and SHR 1.56; 95% confidence interval 1.33–1.83 for a 1 mm increase in the main pancreatic duct diameter) but not with the occurrence of concomitant PDAC [26]. On the same note, Petrone et al. found, in their multivariate analysis, that a dilation of more than 5 mm of the MPD (OR = 24.48, *p* = 0.004) was the most important risk factor for the malignant transformation of BD-IPMN [27]. Finally, Gausman et al. followed patients with asymptomatic IPMN without worrisome features with a median follow-up of 44 months [28]. Of the patients, 48% had a cyst size increase, 12% developed worrisome features, and 2% developed HGD/cancer. The presence of large cysts (>2 cm), a multifocal nature, having prostate cancer, and being a smoker were the main risk factors for the development of alarming symptoms, whereas being male, having a history of diabetes, and recent weight loss were related to development of HGD/cancer [28].

### 2.3. Molecular Classification

KRAS, a known membrane-bound oncogene, is positioned on chromosome arm 12p and usually experiences mutations primarily on codon 12 but also on codons 13 and 61 [29,30]. Studies are in discordance regarding the frequencies of these mutations, which appear to range between 38.2% and 100% [31,32]. This wide array is partially caused by the difference in sensitivity of the chosen screening method. Furthermore, given that the frequency of these mutations is high in all grades, it can be supported that there is no notable differential expression between them. In general, the expression of KRAS can be measured via three different approaches: through analysis of surgical specimens, peripheral blood, and pancreatic exocrine products [30]. KRAS measurements obtained through the last of these approaches appear to be highly unspecific, since levels of KRAS in pancreatic juice are also increased in chronic pancreatitis and other inflammatory diseases [33]. Moreover, KRAS expression is changed both in the very center of the tumor as well as in the adjacent-to-the-tumor area, and in other IPMNs [33]. Okoboyashi et al. noted that there was significant difference in the main duct diameter for patients with and without KRAS mutations, further supporting that these mutations could be correlated with increased mucin secretion [34]. Furthermore, the highest frequency of KRAS mutations was shown among the pancreatobiliary subtype—47% to 86%—while the lowest was shown among the intestinal and oncocytic subtypes—21% to 58% and 0% to 17%, respectively [35]. In addition, it is to be mentioned that KRAS testing is extremely valuable for the malignant transformation of IPMNs in cases where CEA levels are low [36,37]. Nevertheless, given the previously noted non-differential KRAS expression among the different stages of dysplasia, the prevalence of KRAS mutations throughout different IPMN subtypes, and the insignificance to the survival outcomes in patients with IPMN, it can be doubted that knowing the KRAS status would be of significant prognostic and clinical value [35,38,39].

GNAS, another oncogene of interest, is located on the q arm of chromosome 20 and is commonly affected by mutations at the codon 201 [40]. GNAS-mutation importance stems from the fact that is almost exclusively found in IPMNs [30,41]. These mutations activate the cAMP-PKA pathway, thereby promoting tumorigenesis [42]. They are specifically associated with the intestinal subtype, where they are found 59% to 100% of the time. Regarding other subtypes, this mutation is shown to be present in 44% to 71% of pancreatobiliary and 46% to 55% of gastric subtypes [35]. Interestingly enough, GNAS mutation rates in IPMN with pancreatic adenocarcinoma were recently found to be significantly decreased when compared with cases of IPMN without associated adenocarcinoma [43]. GNAS wild-type and gastric-type IPMNs are significantly associated with adenocarcinoma development [43]. On the other hand, in oncocytic IPMNs, GNAS mutations are detected rarely: less than 17% of the time [35]. Generally, GNAS mutations appear in advanced lesions, and with the KRAS ones, they jointly promote tumorigenesis [44]. Moreover, it was recently shown that mutations in GNAS were more common in colloid-type IPMN with invasive carcinoma features than tubular-type IPMN with such characteristics (89% vs. 32%, respectively; *p* = 0.0003), whereas KRAS mutations were typical of tubular-type versus colloid-type IPMN (89% vs. 52%, respectively; *p* = 0.01). Therefore, as expected, KRAS mutations are also common in the tubular type of intraductal papillary carcinomas, which are indistinguishable from colloid pancreatic adenocarcinoma (cPDAC) [42]. Similarly to KRAS mutations, GNAS mutations did not vary according to the degree of dysplasia, suggesting that mutations in these genes occur early in IPMN carcinogenesis. Colloid carcinoma, associated with IPMN and its intestinal-type pre-invasive precursor, is associated with high frequencies of GNAS mutations. If known preoperatively, the mutation profile may exert great influence on clinical management decisions [30]. Similarly, as reported by Gaujoux et al., GNAS mutations are highly associated with the intestinal-type phenotype (*p* < 0.001), and KRAS were not specific to a particular phenotype. Importantly, IPMNs with GNAS mutations were only of the invasive type in ten cases (11%), whereas eight out of ten (83%) were colloid-type. For invasive lesions, their univariate analysis showed a correlation of GNAS mutations with prolonged survival, but in their adjusted analysis, this association lost its significance, with negative lymph nodes being the only significant association with improved cancer-specific survival in the multivariate model [45].

In trying to comprehensively investigate the somatic mutations that drive tumorigenesis, a great heterogeneity was found among different IPMNs. The lower the grade of dysplasia, the greater the heterogeneity in KRAS/GNAS mutations (*p* < 0.02) was. It was also shown—through whole-exome sequencing—that a single IPMN may contain completely independent clones with different mutation statuses. Additionally, it seems that there is a parallel evolution of other mutations regarding RNF43 and TP53, which typically happen later [46]. In more detail, RNF43 mutations are found in a minority of IPMNs—less than 15%—and are most common among the intestinal type. Despite the fact that they are associated with dysplasia progression, they are not associated with transformation to invasive carcinoma [35,42]. Lastly, mutations like TP53, SMD4, and CDKN2A appear more commonly in invasive neoplasms, suggesting that they are mainly implicated in later stages, like progression to invasiveness, rather than the initiation of IPMN. TP53 mutations are present in more than half of tubular IPMC, whereas SMAD4 and CDKN2A are present in 20% to 40% [42]. Table 1 provides a summary of the genes that are pertinent to IPMNs along with molecular markers and other markers commonly measured from the cystic fluid.

## 3. Diagnostic Assessment of IPMNs

### 3.1. CT/MRI

The initial step in IPMN cyst evaluation is contrast-enhanced MRI with MRCP, with a triphasic CT being a good alternative for patients with contraindications to MRI/MRCP [21]. The purpose of the aforementioned is to investigate for high-risk findings, namely, a cyst > 3 cm, a solid component within the cyst, and main pancreatic duct dilation [47,48]. Another goal of these studies is to assess the disease topography, which may suggest malignant-like involvement of surrounding, or even distant, structures [49,50]. Kashiwagi et al. assessed risk factors of progression noted upon imaging patients with low-risk BD-IPMN, including pancreatic fat content and CT attenuation indices. In their multivariate analysis, the initial cyst size, along with the pancreatic and splenic indices, was crucial in determining disease progression in IPMN [51]. Moreover, with the addition of pancreatic fat content, these signs where highly related to BD-IPMN progression. Taking all the above into consideration, the authors champion that initial cyst size and pancreatic fat content should be added to a potential revision of the Fukuoka guidelines [51]. Zhang et al. evaluated the correlation of CT fish-mouth ampulla sign with the presence of HGD or invasive cancer in patients with pancreatic duct dilatation, with or without IPMNs [52]. In patients with histologically proven MD/MT IPMNs, the CT fish-mouth ampulla sign was found in 18.5% of cases with excellent specificity and PPV (100%) and very good NPV (79.8%) when compared to appropriately matched controls without IPMN [53]. On the contrary, the CT fish-mouth ampulla sign was not related with the presence of HGD or invasive features [52]. Overall, a combination of contrast-enhanced studies is recommended, as have Ohno et al., for the follow-up of these patients. In more detail, there is support for the idea that multidetector CT and MRI should be the gold standard for the detection of mural nodules with or without solid components, whereas EUS is the best for further characterization of these findings and potentially performing further necessary testing, like FNA, if needed [22].

### 3.2. Endoscopic Ultrasound (EUS)

EUS is both a great imaging and diagnostic tool. The sensitivity and specificity of malignancy-suggesting findings are yet to be determined; these findings consist of main duct dilation of more than 7 mm in MD-IPMN, a cystic lesion >30 mm with irregular thick septations in BD-IPMN, and a mural nodule of more than 10 mm in both IPMN types [54]. Ohno et al. identified predictors of malignant transformation using contrast-enhanced endoscopic ultrasound (CE-EUS). Papillary (88.9%) and invasive (91.7%) mural nodules and symptomatic IPMNs were more frequently associated with malignant lesions. The existence of type III or IV nodules reached a sensitivity of 60% and a specificity of 92.9% for malignancy [55]. In the same frame, Pais et al. showed that patients with malignant IPMNs had a dilated main pancreatic duct (*p* = 0.0001), solid component (*p* = 0.0001), ductal filling defects (*p* = 0.03), or thickened septa within cysts (*p* = 0.02). The sensitivity, specificity, and accuracy of EUS-FNA for the diagnosis of malignancy were 75%, 91%, and 86%, respectively [56]. Another study evaluating the predictive role of preoperative EUS for BD-IPMNs showed that only the branch-type diameter was significantly different in low- versus high-risk lesions for malignancy (31.5 mm vs. 41.9 mm, *p* = 0.0225). Mural nodule diameter and its width diameter were significantly correlated with low- and high-risk IPMNs [57]. Interestingly, 70 studies that used CE-EUS to characterize the characteristics of mural nodules were used in a meta-analysis that concluded that the size of mural nodules is highly associated with the progression of IPMNs both to having high-grade dysplasia and ultimately to invasive carcinoma, but no specific size cutoff was proposed. Nevertheless, the meta-analysis concluded that CE-EUS is probably the best for studying and characterizing a potential mural nodule and, thus, accurately aiding in the prediction of malignancy [58].

In patients with BD-IPMNs, EUS features including mural nodules, main pancreatic duct involvement, and suspicious or positive cytology were highly specific for malignant transformation. EUS was also superior in identifying mural modules that were missing in CT/MRI in 28% of the cases. A main pancreatic duct size of 5 to 9 mm was the strongest predictive factor of malignant transformation of BD-IPMNs [59]. Septal thickness (ST) has been proposed as an indicative sign of malignant BD and MT-IPMN. More specifically, the discrimination that ST offers is different when measured from histological specimens versus EUS versus CT (C-statistics: 0.74, 0.70, and 0.56, respectively). In a multivariate analysis, the ST was found to be greater than 2.5 mm and had an OR of 3.51 (*p* = 0.003) and having a mural nodule of more than 5 mm in height resulted in an OR of 3.36 (*p* = 0.003), making them independent predictor factors for the malignant nature of IPMNs [59,60].

### 3.3. Cyst Fluid Analysis

#### 3.3.1. Carcinoembryonic Antigen (CEA)

There are mixed results regarding the value of CEA in predicting IPMN with underlined malignancy. On the one hand, Kucera et al. found that this measurement has poor prediction value, whereas Maire et al. suggest that a cutoff of 200 ng/mL or more has around 90% sensitivity for underlying malignancy in IPMNs [61,62]. In the same direction, Koker et al. found that a cutoff value of >100 ng/mL has a 100% negative predictive value for discerning high- from low-risk mucinous cystic neoplasms [63]. Moreover, Pitman et al. suggest that a bigger—more than a 10-fold—threshold for CEA (2.500 ng/mL) or the presence of atypical epithelial cells works better for predicting malignancy in small-branch IPMNs when compared to the established guidelines [64]. Hayakawa et al. reported that a CEA level of more than 97 ng/mL in pancreatic juice had a sensitivity of 45% and a perfect specificity of 100% for detecting malignancy. The specificity was similarly high across the different types of IPMNs, but on the other hand, sensitivity was better in MD-IPMN. The significance of increased CEA was highlighted by the fact that patients with higher levels had a much higher 5-year malignant transformation rate: 69% versus 0%, *p* < 0.001 [65]. Another study assessed biochemical and tumor marker levels in IPMN cystic fluid. It showed that a CEA level greater than 200 ng/mL and a CA72.4 level over 40 U/mL both had less than 50% sensitivity for establishing IPMN diagnoses. On the contrary, the levels of the biomarkers CEA, CA 19.9, and CA 72.4 had better performance when used to differentiate benign from malignant processes. Namely, CEA levels of more than 200 ng/mL had a sensitivity of 90% and a specificity of 71%, and the same metrics for CA 72.4 of more than 40 U/mL were 87.5% and 73%, respectively [62]. Overall, according to Wang et al., CEA levels have low sensitivity but high specificity for invasiveness and malignancy in IPMN, whereas CA 19-9 appears to be the more appropriate metric for differentiation between benign and malignant IPMNs [36]. Another study showed that a CEA of >30 ng/mL—as measured in the pancreatic exocrine product—was an independent factor associated with malignancy in BD-IPMN with a PPV of 100% and a NPV of 96.3% [23]. The accuracy of worrisome features for classifying malignant IPMN was increased by the addition of pancreatic juice cytology but the accuracy of HRS was not [66]. Overall, CEA analysis only is insufficient, and molecular analysis of the cystic fluid seems necessary for appropriate risk stratification [67].

Cyst fluid amylase indicates pancreatic duct communication, and it is commonly used to differentiate pancreatic cystic neoplasms. Amylase is most commonly increased in pseudocysts rather than IPMNs, and a cutoff of more than 479 U/L has a sensitivity and specificity of 73% and 90%, respectively, for distinguishing between the two pathologies [68]. On the contrary, cyst amylase levels of <250 U/L virtually exclude the diagnosis of pseudocysts [69].

#### 3.3.2. Molecular Profiling

Other important genes are the Mucins, with MUC1 and MUC2 being specific for aggressiveness, which are also displayed on Table 1 [70,71]. Regarding IPMNs, MUC1 is present in 90% of tubular-type invasions, followed by malignant IPMN with 35.8% and borderline IPMNs with 8.6% prevalence [72,73]. MUC2 importance stems from the fact that it not expressed by normal pancreatic tissue, but it can be found in IPMNs of the intestinal type and in colloid carcinomas [31,70,73]. On the other hand, among 1% of ordinary PDACs, only 1% of the samples express MUC2 [70]. MUC2 expression is believed to be correlated with coexisting gastrointestinal malignancies [74]. In invasive carcinomas arising from IPMNs, colloid carcinomas are characterized by MUC2, while lacking MUC1 expression. Tubular carcinomas, on the other hand, usually express MUC2 (60%) and lack MUC1 (1%). Maker et al. reported that MUC2 and MUC4 were produced more by high-risk IPMNs when compared to low-risk ones [69] (10 ± 3.0 ng/mL vs. 4.4 ± 1.2 ng/mL, *p* = 0.03; 20.6 ± 10.6 ng/mL vs. 4.5 ± 1.4 ng/mL, *p* = 0.03, respectively). Additionally, the serum levels of MUC5AC were more elevated in high-risk patients (19.9 ± 9.3 ng/mL vs. 2.2 ± 1.1 ng/mL, *p* = 0.04). Furthermore, the intestinal subtype was significantly correlated with increasing dysplasia grade and increased MUC2 concentration in cystic fluid (13.8 ± 6.5 ng/mL vs. 4.1 ± 0.9 ng/mL, *p* = 0.02) [75]. Das et al. developed a monoclonal antibody against a colonic epithelial phenotype called mAb Das-1. Das-1 appears to have pronounced reactivity in both EUS-FNA obtained cystic fluid and in histological specimens from resected high-grade IPMNs, with a sensitivity and specificity of more than 85% [76]. mAb Das-1 was preferentially produced in higher-grade lesions [76] but way less reactive in IPMN with LGD/IGD (3%), which most frequently represent indolent BD-IPMN [77].

Schneider et al. showed that prostaglandin E2 (PGE2) levels in high-grade IPMNs were significantly higher compared to low-/moderate-grade IPMNs (*p* < 0.0016). They also noticed that if the PGE2 threshold was set to 1.1 pg/μL, the sensitivity, specificity, and overall accuracy in the detection of high-grade dysplasia were 63%, 79%, and 71%, respectively. Importantly, in a subset of patients with CEA levels greater than 192 ng/mL, a PGE2 threshold of 0.5 pg/μL produced 78% sensitivity, 100% specificity, and 86% accuracy for the detection of HGD/invasive IPMN [78].

The presence of HGD or invasive cancer seems to generate a proinflammatory microenvironment in IPMN. It seems that Interleukin IL-1β, IL-5, and IL8 expressions are significantly increased in the cyst fluid from high-risk patients, whereas no significant differences are found in other proinflammatory cytokines like IL2, IL4, IL10, IL12, IL13, TNF-α, or IFN-γ between high- and low-risk patients [79]. In one study, the combination of elevated PGE2 and IL-1β achieved an AUC of 0.789, with a specificity of 89% and a PPV of 82% for HGD/invasive IPMN [80].

Finally, DNA sequencing performed in pancreatic cyst fluid can identify important mutated genes that can help in differentiating cyst types [81]. In patients with IPMN, KRAS and GNAS were the most common mutations, forming as high as 76% of the cases. Other mutations in CDKN2A, SMAD4, and TP53 are typical of mucin-producing cysts, associated with an odds ratio between 2.8 and 7.2 of HGD or early cancer [82]. Similarly, mutations in BRAF, CDKN2A, CTNNB1, GNAS, KRAS, NRAS, PIK3CA, RNF43, SMAD4, and TP53 were found in IPMN-derived cancers [82]. Interestingly, somatic mutations in another gene—STK11/LKB1—can happen; germline mutations of the same gene are implicated to the pathogenesis of Peutz–Jeghers syndrome [83]. The combination of these mutations with clinical and radiographic data achieved a 90–100% sensitivity and 92–98% specificity in accurately diagnosing pancreatic cyst neoplasms and facilitated the reduction of patients undergoing unnecessary operations by 91% [84].

#### 3.3.3. MicroRNA

MicroRNAs are very promising in the development of biomarkers in the diagnosis and management of IPMN. In short, MicroRNAs are small single-stranded RNA molecules that help in gene expression regulation [85], and their abnormal expression is noticed both in PDAC and its precursor lesions [86]. There is differential expression of MicroRNA-21 and MicroRNA-155 between invasive IPMNs and non-invasive IPMNs and normal pancreatic tissue, being higher in the former group [86,87]. Other important correlations include MicroRNA-708 with malignant transformation and MicroRNA-21 with shorter survival rates [87,88]. On the contrary, the downregulation of MicroRNA-200c and MicroRNA-141 is associated with more invasive disease [89]. Lastly, the downregulation of miR-99a has a high discrimination ability, with a C-statistic of 0.87, for high-risk (HGD and invasive) and low-risk lesions [90].

Long non-coding RNAs (lncRNAs) have been reported to play a regulatory role in the invasion and metastasis of PDAC. More specifically, lncRNA TUSC7, LINC00052, and lncRNA CASC2 are shown to modulate miR-371a-5p, miR-330-3p, and miR-21, respectively, to suppress pancreatic cancer cell lines [91,92]. Recently, it was shown that lncRNAs may play a role in IPMN carcinogenesis. LncRNAs such as HAND2-AS1 and CTD-2033D15.2 were negatively correlated with IPMN carcinogenesis (*p* = 0.0026 and 0.000147), while lncRNA-TFG was positively correlated with invasive disease (*p* = 3.99 × 10^−8^) [93].

## 4. Nomograms and Predictive Models

Recently, there have been several predictive models for HGD and malignancy potential in IPMNs. Most recently, He et al. created a prediction model using random forest to discern between three different classes—LGD, HGD/pT1, and invasive carcinoma—achieving an accuracy of 96% [94]. Attiyeh et al. proposed another model of predicting the development of HGD or invasive cancer in patients with IPMN using a large multi-institutional dataset with a 10-year follow-up period. Patients with MD-IPMN had more than double the change of high-risk disease compared BD-IPMN (71% vs. 29%, *p* < 0.001). Furthermore, the presence of jaundice was almost exclusively associated with high-risk disease (98% of the patients), and lastly, a cyst size > 3.0 cm, a solid component/mural nodule, pain symptoms, and weight loss were significant predictors of patients with high-risk disease [95].

Al Efishat et al. proposed predictive models on the natural history of IPMN by incorporating cystic fluid markers in clinical nomograms. The cyst fluid markers that were used were MMP9, CA72-4, sFASL, and IL-4, which were overproduced in high-risk IPMN patients (*p* < 0.05). Two predictive models based on preselected combinations of cystic fluid markers were created and then integrated into an already-established nomogram based on clinical variables. Ultimately, this integration increased discrimination compared with either the cystic fluid models or nomogram alone (c-indices of 0.84 and 0.83, respectively) [96].

A recent study further added to the accuracy of current predictive models of IPMN’s progression to cancer, by combining clinical data, qualitative radiographic reports, and analysis of cyst fluid. This model achieved an area under the curve (AUC) of 0.74. The addition of quantitative imaging using CT scan data improved the AUC to 0.83. Interestingly, the positive and negative predictive values were 75% and 86%, respectively, suggesting that low- and high-risk disease can be predicted prior to surgery [97]. The AUC further improved to 0.91 when tumor-associated neutrophils (TANs) measured by a pathologist on the resected specimen were included. Interestingly, the addition of TANs improved the sensitivity of all models, suggesting that the risk of false negatives can be mitigated at the time of resection with this additional information. Finally, the addition of cystic fluid analysis to the latter model (radiomics + TAN + clinical data) provided a high AUC (0.98), a positive predictive value of 87.5%, and a negative predictive value of 100% [97]. An overview of the above is provided in Table 2.

## 5. Management of IPMN

### 5.1. Risk Stratification Approaches (Consensus Guidelines)

**Figure 2:**
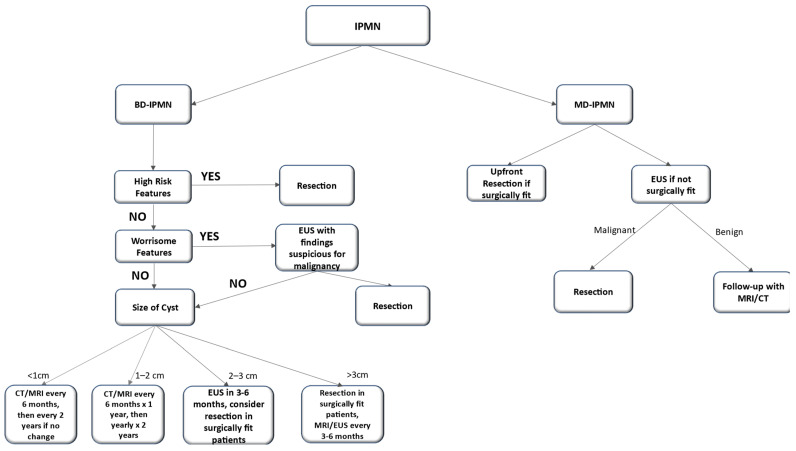
Algorithm for the management of IPMNs.

Ideally, early surgery of pancreatic lesions at the stage of HGD can prevent malignant transformation. Finding reliable predictors for HGD and invasive cancer is the current focus for clinicians. As we have already discussed, there are several clinical and radiological characteristics predictive of malignant transformation in IPMN. According to both the 2017 IAP [98] and the 2018 European guidelines (Figure 2) [99], jaundice, an enhancing mural nodule ≥ 5 mm, a solid component, positive cytology for HGD or invasive cancer, and a dilated pancreatic duct ≥ 10 mm are all highly predictive of the progression of IPMN, and therefore, they cumulate to an indication for surgical resection, especially in surgically fit patients. According to both the aforementioned guidelines [96,97], acute pancreatitis caused by IPMN, an enhancing mural nodule < 5 mm, a dilated pancreatic duct between 5 mm and 9.9 mm, and an increased level of serum carbohydrate antigen 19-9 without jaundice, are also indications for surgical intervention. Segmental pancreatic dissection with regional lymphadenopathy remains the preferred surgical treatment even though IPMNs affect the whole pancreas [96,100]. This reduction, rather than excision, of the whole disease is the main culprit behind the high recurrence and progression rates of disease in the remnant gland. Therefore, all the guidelines propose that the experience of the surgeon and a multidisciplinary approach form the cornerstone of IPMN management. Finally, in addition to the accumulated experience that a multidisciplinary team has to offer, radiomics seems to also have a promising role in the future of guideline formation for the risk stratification and management of patients with IPMN [101]. Namely, studies like that performed by Chakraborty et al. showcase that a combination of high-order radiological signs with clinical variables is important in the quantification of risk for high-grade dysplasia [102]. This importance lies in the collection of high-volume, high-quality radiological images that will allow such models to accurately classify dysplasia and therefore guide treatment guidelines [101].

Another set of international guidelines—the Kyoto guidelines—present a revision of the previous consensus rules. Interestingly, its new algorithm introduces EUS as an imaging modality for characterizing high-risk stigmata both visually and through the cytology obtained. One of the highlights is that the surveillance of small BD-IPMN over 5 years may no longer be necessary. Overall, these guidelines also address pathological aspects, such as the role of cystic fluid markers, among others [13].

### 5.2. Approach to Radiographic Surveillance

In patients with a 5 to 9 mm MD-IPMN who do not undergo surgery, MRCP or CT scan within one year is recommended. If the duct is stable on repeat imaging, lengthening of the surveillance interval to every two years is recommended. In patients with a pancreatic duct < 5 mm, follow-up MRCP or CT in two years is recommended [103]. The optimal surveillance approach is unclear. For MRI, the American Gastroenterological Association suggests that the first round of surveillance should be within 1 year post diagnosis and then in 2-year intervals for cysts without size changes or development of worrisome characteristics [104]. On the other hand, the Sendai consensus guidelines suggest a more aggressive and variable surveillance strategy with intervals based on the size of the IPMN; In short, more aggressive-looking lesions should be followed by EUS versus CT for less aggressive ones, and the intervals shorten or become longer as necessary [98,105].

More recently, the optimal radiographic surveillance strategy for patients with BD-IPMNs has been further elucidated. In their multi-center international cohort study, Han et al. propose that optimal surveillance should be based on the size of the cyst when first found. Their proposal is that, regardless of their size, the initial round of surveillance should be performed at 6 months, and then, the cysts should be classified into three groups based on size—<2 cm, 2–3 cm, and >3 cm in diameter—and the best surveillance interval, thereafter, is every 1.5 years, 1 year, and 6 months, respectively. Lastly, surveillance was deemed redundant in patients who have had stable small (<2 cm) cysts for 5 years [106].

### 5.3. Approach to Surgical Resection

**Figure 3:**
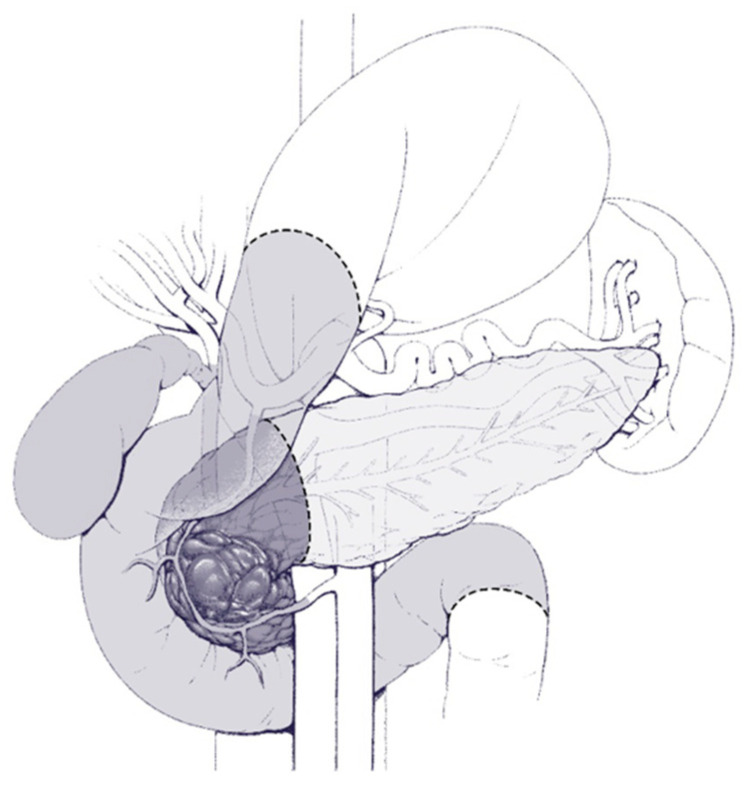
Illustration of the extent of resection during a typical Whipple procedure.

**Figure 4:**
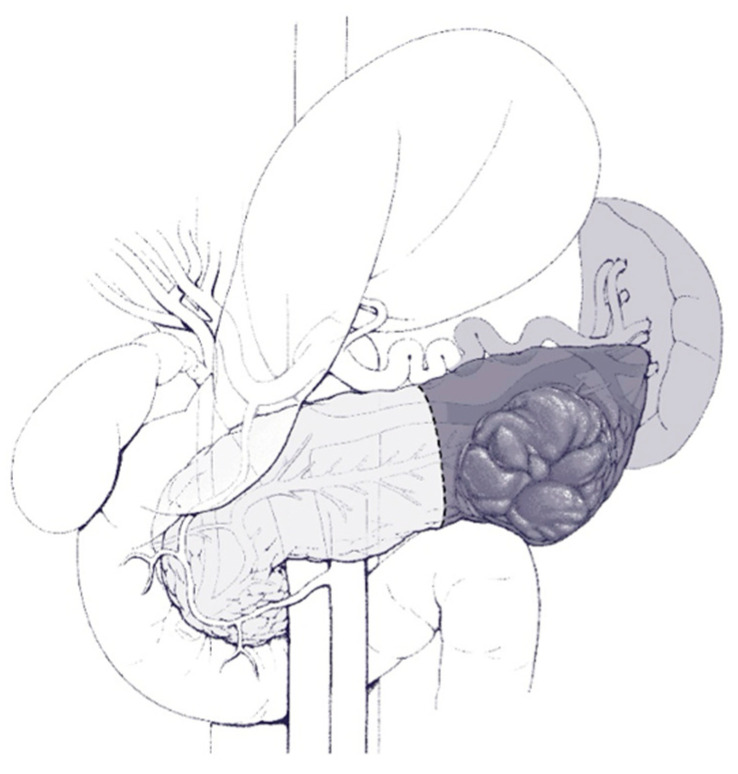
Illustration of the extent of resection during a typical distal pancreatectomy.

**Figure 5:**
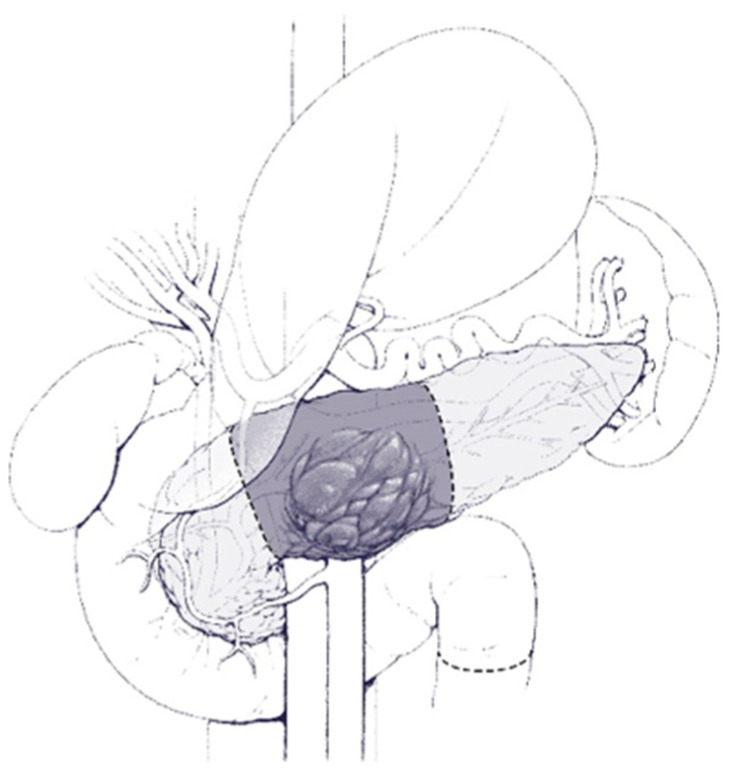
Illustration of the extent of resection during a typical central pancreatectomy.

Different surgical procedures are indicated according to the location of the mass. In short, and as shown in Figure 3 and Figure 4, lesions on the head of the pancreas require the Whipple procedure, whereas lesions in the body/tail of the pancreas require distal pancreatectomy, and they can be performed via either open or minimally invasive means [107,108,109,110]. Less extensive resections, such as central pancreatectomy, can be performed, which consists of a parenchyma-sparing technique as shown in Figure 5.

Surgical resection of IPMN is associated with a morbidity of 20–40% and a perioperative mortality of 1–3% for pancreatoduodenectomy and <1% for distal pancreatectomy in experienced centers [111,112,113,114]. The most common complications from Whipple procedures, in descending order of frequency, include exocrine insufficiency, pancreatic fistula, and new-onset diabetes mellitus, having frequencies of 40%, 15%, and 10%, respectively [115]. For distal procedures, the overall complication rate is around 25%, including pancreatic fistula in 15%, new-onset diabetes mellitus in 10%, and exocrine insufficiency in 20% [116]. Lastly, central pancreatectomy has excellent post-surgery endocrine function, but there is a higher risk of overall morbidity (50%) and pancreatic fistula (35%) [117].

In symptomatic patients with BD-IPMN, the indications for surgical resection include high-risk stigmata. It is worth noting that patients with two relative indications for surgery have an almost five-time greater risk of having invasive carcinoma in comparison to those with one; the associated risks are 24% and 5.7%, respectively [118]. The standard approach is segmental pancreatectomy with lymphadenectomy or enucleation depending on the location of the lesion. Limited or focal non-anatomic resection can be considered in cases where preoperative workup or intraoperative findings are not concerning for malignancy. There is emerging literature on the role of enucleation for BD-IPMN [119,120]. On the contrary, due to the lack of lymphadenectomy, this treatment might lead to an inferior oncological approach in cases of undiagnosed HGD or invasive cancer harboring in BD-IPMN [121]. Another approach reported in the literature is EUS-guided pancreatic cyst ablation using ethanol and/or paclitaxel [122,123,124]. EUS-guided cyst ablation is a good substitute, since it enables organ preservation with largely intact endocrine and exocrine function [125]. However, the main concern is the associated 2–10% morbidity rate, presumably attributed to the use of ethanol [126]. The inability to obtain a biopsy is another downside of this approach; thus, the efficacy of EUS-guided ablation is not well established [126,127]. While the above minimally invasive techniques may be appropriate for benign-looking BD-IPMNs, the risks of not obtaining histopathological confirmation should be considered given that approximately one in three IPMNs undergoes transformation to carcinoma before resection [128]. For multifocal BD-IPMN, the number of lesions positively correlate with the risk of invasive disease. But this is not always the case, since some data support that the presence of symptoms in unifocal BD-IPMN may change the direction of the relationship [129]. The treatment strategy comprises segmental pancreatectomy for high-risk lesions, with an attempt at avoiding total pancreatectomy if the disease is anatomically limited [105].

In MD-IPMN, resection is typically indicated in all radiologically or endoscopically confirmed MD-IPMN given the high risk of invasive and malignant disease. The greater challenge in MD-IPMN is the selection of the appropriate operation due to inadequate preoperative localization of the area with HGD or invasive disease [130]. When an invasive lesion is identified, segmental pancreatectomy should be performed. In the absence of an invasive lesion, if there is a discrete radiographic distribution of the disease in one portion of the gland, segmental pancreatectomy is also recommended, accompanied by intraoperative frozen section to rule out HGD at the resection margin and exclude the need for margin re-excision. Unfortunately, the accuracy of frozen section is limited with PPV to about 50% and NNV to about 75% [131].

The decision to proceed with total pancreatectomy in patients with IPMN is challenging because it mandates careful patient selection, and it is related to considerable long-term morbidity and inevitable endocrine and exocrine insufficiency [132]. According to a SEER-based study performed by Wang et al., total pancreatectomy is not shown to significantly ameliorate cancer-related survival when compared to the Whipple procedure. However, it may be beneficial in patients with T4 disease or AJCC stage III, while the less radical Whipple procedure may be better for older patients and cases with poorly differentiated disease [133]. In patients with BD-IPMN, total pancreatectomy should only be considered in cases of high-risk lesions with diffuse whole-gland involvement [132]. In patients with MD-IPMN where there is a diffuse ductal dilation without focal lesions on imaging mandated as part of work-up with ERCP/EUS to exclude occult malignancy [105]. In the absence of localized invasive disease, total pancreatectomy should be considered.

## 6. Management of Pancreatic Remnant

There are emerging data on the management of pancreatic remnant after IPMN resection, given that recurrence risk is relatively high, and the type can range from benign IPMN to PDAC [134,135,136]. Unfortunately, there is no consistent definition for disease progression within the remnant. Thus, a more collective definition has been deemed necessary to ensure the accuracy of future post-surgery surveillance studies [137].

Regarding risk factors for progression within the remnant, being symptomatic, a body/tail location, a main duct of more than 10 mm in diameter, and a primary lesion having high-grade dysplasia or that is an invasive pancreatic mucinous carcinoma (IPMC) are the four independent risk factors that Hirono et al. suggest for postoperative malignant conversion of IPMN or postoperative PDAC. The associated hazard ratios were reported to be 1.99 (*p* = 0.005), 3.88 (*p* < 0.001), 1.90 (*p* = 0.021), and 3.20 (*p* < 0.001), respectively [138]. An earlier study from Al Efishat et al. had also showed that distal progression was associated with an increased risk of progression (*p* < 0.001) [135].

Targeted next-generation sequencing (NGS) technology has been used to provide insights on the potential mechanisms of local disease progression in the pancreatic remnant. Pea et al. performed NGS on genes commonly mutated in IPMN and PDAC in patients who developed disease progression after surgery. The progression of pancreatic remnant disease has been attributed either to residual microscopic disease at the resection margin or the intraparenchymal spread of neoplastic cells in the parenchyma of the remnant that leads to genetically similar but anatomically different diseases or unrelated genetically multifocal diseases with distinct lesions [139]. A family history of pancreatic adenocarcinoma (*p* = 0.027) and high-grade dysplasia (*p* = 0.003) have been reported as independent risk factors for future progression on the remnant pancreas [139].

Following surgery, the risk of developing a subsequent recurrence in the remnant pancreas is around 5% [140]. In such patients, yearly follow-up with MRCP or CT is a reasonable surveillance approach [135], and the criteria for a second surgery are the same as those for the first. Regarding patients with curative resection of invasive carcinoma arising from an IPMN, studies suggest that the risk of IPMN recurrence is 25 to 50% [140], and surveillance every six months is appropriate [134,141].

## 7. Adjuvant Therapy

Adjuvant chemotherapy with or without radiation is a new therapeutic option in patients with IPMN. The current literature shows equivocal results. In a retrospective cohort study by Rodriguez et al., patients receiving adjuvant chemoradiation after IPMN resection were younger, presented at higher T and N stages and had more perineural and lymphovascular invasions compared to patients receiving surgical treatment only. Interestingly, the median overall survival (OS) was 134 months in the surgery-alone group and 65 months in the adjuvant-treatment group without reaching statistical significance (*p* = 0.052). In their multivariate analysis, adjuvant treatment was not associated with improved OS [hazard ratio (HR) = 1.03 (0.52–2.05)]. In patients without nodal disease (N0), compared to surgery alone, adjuvant therapy was associated with a worse median OS (65 vs. 167 months, *p* = 0.03), whereas in node-positive patients, there was a non-significant improvement (50.5 vs. 20.4 months, *p* = 0.315) [142]. In addition, in IPMN patients with an AJCC TNM stage past or equal to II, positive lymph nodes, positive margins, and poorly differentiated tumors demonstrated improved OS with adjuvant therapy [143]. Adjuvant therapy seems to have no significant effect on the survival of patients with less aggressive tumors, and in general, patients with invasive IPMN had improved risk-adjusted OS compared with those who had PDAC (HR, 0.73; 95% CI, 0.68–0.78; *p* < 0.00001) [143]. In the same view, a Surveillance, Epidemiology, and End Results (SEER) analysis showed that adjuvant RT after IPMN resection offered no survival benefit compared to surgery alone (5-year OS: 23.5 months versus 23.5 months, *p* = 0.23). Univariate predictors of survival were nodal involvement, T4-classified tumors, and a poorly differentiated tumor grade (all *p* < 0.05) [144]. In a propensity-score-adjusted analysis, adjuvant RT was associated with an improved 5-year OS (HR: 0.73, *p* = 0.014) among patients with LN involvement, although further analysis using T-classification demonstrated no survival differences among patients with T1/T2 disease. On the contrary, patients with T3-/T4-classified tumors had improved cancer-specific survival (HR: 0.71, *p* = 0.022) but no difference in OS (HR: 0.76, *p* = 0.06) [144]. Overall, due to the conflicting results regarding the benefit of the AC, it is suggested that a multidisciplinary and personalized approach be implemented, and adjuvant chemotherapy be reserved for patients with nodal disease or its later stages [145].

## 8. Conclusions

There is no reliable method of discerning between low-risk and high-risk IPMNs with current laboratory, endoscopic, cytologic, and imaging modalities. Surgery remains the mainstay of treatment in eligible patients. Current guidelines recommend resection for MD-IPMN patients and observation for the majority of BD-IPMN. Performing opportune resection of HGD lesions before their progression to invasive disease and avoidance of overtreating benign lesions with minimal dysplasia with an invasive surgery is the optimal clinical scenario. Therefore, the development of preoperative models able to discern HGD in IPMN patients is needed. Low-risk patients should be managed with nonsurgical treatment options (typically MRI surveillance), while high-risk patients would undergo resection, possibly prior to the formation of invasive disease. Current research is evolving in multiple directions. First, there is an ongoing effort to identify reliable markers for predicting malignant transformation of IPMN, mainly focusing on genomic and transcriptomic data from blood, tissue, and cystic fluid. Also, multimodal models of combining biomarkers with clinical and radiographic data seem promising for providing robust and accurate answers on risk levels for IPMN patients.

## Figures and Tables

**Table 1:** Overview of the function of the molecular markers.

Marker	Category	Function
CEA	Common Markers	Obtained both from serum and cystic fluid; has low sensitivity and high specificity for malignant and invasive IPMNs
CA 19-9		Obtained both from serum and cystic fluid; aids with invasive from benign IPMNs differentiation
KRAS	Oncogenes	Around 50% of all IPMNs have this mutation; can be measured in peripheral blood, excised tissue, and exocrine pancreatic products
GNAS		Around 60% of all IPMNs have this mutation; even more common in a subset of advanced lesions
MUC1		Usually obtained from the cystic fluid via EUS; increased expression associated with adenocarcinoma derived from IPMNs
MUC2		Usually obtained from the cystic fluid via EUS; in intestinal-type IPMNs and colloid adenocarcinomas
miR21, miR155, miRNA-708	MicroRNAs	Commonly measured in resected pancreatic tissue; upregulation in invasive IPMNs
IL-1b	Cyst Inflammatory Markers	Higher levels in the cystic fluid from patients with HGD/cancer
IL-5 and IL-8		Higher levels in the cystic fluid from patients in the high-risk group

**Table 2:** Overview of the prediction models.

Author	Main Statistical Method	Objective	Results	Additional Considerations
He et al., 2023 [94]	Random forest	Creation of model that differentiates between LGD, HGD, and invasive carcinoma	96% accuracy in the testing dataset	Most important risk factors: mural nodule size, MD diameter, CA19-9 levels, common bile duct dilation
Al Efishat et al., 2018 [96]	Logistic regression modeling	Investigation of whether cyst fluid markers increase the accuracy of clinic-only predictive models	Combination of clinical and cystic fluid characteristics increase the discrimination of the prediction models	MMP9, CA72-4, sFASL, and IL-4 overexpressed in high-risk IPMN
Attiyeh et al., 2018 [95]	Logistic regression modeling	Prediction of low-risk or high-risk disease	C-index in the validation dataset was 0.81	Jaundice, cyst size > 3.0 cm, presence of mural nodule, pain symptoms, and weight loss associated with high-risk disease
Harrington et al., 2020 [97]	Random forest and logistic regression	Combination of cystic fluid with radiomics for the prediction of disease progression	AUC (only cystic fluid variables) of 0.74; AUC (addition of radiomics) of 0.88	Quantitative image analysis, important for accurate predictions
